# Automatic single‐isocenter multiple‐target cranial stereotactic treatment plan optimization via planning system scripting

**DOI:** 10.1002/acm2.70832

**Published:** 2026-09-30

**Authors:** Kurtis H. Dekker, Jennifer A. Everaars

**Affiliations:** ^1^ Medical Physics Department Cancer Centre of Southeastern Ontario Kingston Health Sciences Centre Kingston Ontario Canada; ^2^ Department of Oncology Queen's University at Kingston Kingston Ontario Canada; ^3^ Department of Physics, Engineering Physics, and Astronomy Queen's University at Kingston Kingston Ontario Canada; ^4^ Radiation Therapy Department Cancer Centre of Southeastern Ontario, Kingston Health Sciences Centre Kingston Ontario Canada

**Keywords:** SIMT, TPS scripting, plan quality, planning automation

## Abstract

**Background:**

Single‐isocenter multiple‐target stereotactic radiation treatments delivered on C‐arm linear accelerators are increasingly common due to practical advantages in both accessibility and speed compared to multiple isocenter treatments. Optimization of high‐quality treatment plans can be a time‐consuming process requiring substantial manual effort. In our institution, the planning workflow for these treatments was entirely manual and involved the generation of optimization structures and repeated recalculation and input of optimization objectives during the iterative optimization process.  Although commercial automated planning solutions are available, their implementation may impose operational constraints, including reliance on specific immobilization and image/surface guidance hardware, motivating the development of an immobilization‐independent automation tool for cranial stereotactic treatment plan optimization.

**Purpose:**

To develop and implement a treatment planning optimization tool for multiple‐target cranial stereotactic treatments in the Varian Eclipse treatment planning system, to reduce manual planner input and planning time while improving plan quality.

**Methods:**

A software tool was written using the Varian Eclipse Scripting Application Programming Interface to automatically generate target‐specific ring structures and facilitate the iterative process of plan generation. Twenty cases were retrospectively re‐planned with this tool and compared with the corresponding clinical plans to evaluate plan quality using qualitative and quantitative metrics of conformality and complexity. Automated plans were verified using portal dosimetry to ensure clinical deliverability. For ten cases, a timing study was performed to compare optimization time between the software tool and manual re‐optimization.

**Results:**

The automatic optimization tool produced plans with similar modulation and complexity, but consistently lower dosimetric falloff metrics (R_50%_ and Paddick Gradient Index) than the corresponding clinical plans. All plans passed patient‐specific QA (portal dosimetry) following institutional practice. For the ten cases included in the timing study, the software had a mean runtime of 5.8 min (range: 2–12 min), with variation depending on case characteristics such as number of targets and arcs employed. In comparison, manual plan generation required 21.5 min on average (range: 4–49 min).

**Discussion:**

The automated planning tool produced clinically acceptable, deliverable plans with better dose falloff compared to the previous manual planning approach. The tool is estimated to save an average of 15 min of optimization time per plan.

**Conclusions:**

A treatment planning optimization tool has been developed that provides improvement in both dosimetric plan quality and treatment planning efficiency for single‐isocenter multiple‐target cranial stereotactic treatments.

## 1 INTRODUCTION

Stereotactic radiation therapy is commonly used to treat cranial metastases to high doses with steep dose gradients in highly conformal plans.^1^, ^2^ C‐arm linac‐based single‐isocenter, multiple‐target (SIMT) stereotactic treatments are attractive, and increasingly common, due to reduced treatment times compared to single‐isocenter, single‐target treatments and/or treatments delivered by dedicated stereotactic treatment units. Additionally, adopting C‐arm linac treatment of cranial metastases improves patient access to care at small to mid‐sized centers without dedicated stereotactic equipment. There are several methods described for the planning of SIMT treatments, including both manual optimization strategies^3^, ^4^, ^5^, ^6^, ^7^, ^8^, ^9^ and partially‐automated vendor‐provided solutions, such as BrainLab's Elements^10,11^ (Brainlab SE, Munich, Germany) or Varian's HyperArc^5^, ^12^, ^13^, ^14^ (Siemens Healthineers, Erlangen, Germany). In addition, various authors have described in‐house scripting tools designed to automate some or all parts of the SIMT planning process.^15^, ^16^, ^17^, ^18^ There is some variety in the strategies used by both manual and automated tools to optimize VMAT SIMT plans, but in general the approaches rely on the use of optimization rings/shells around target volumes^3^, ^6^, ^7^ or on normal tissue objectives^12^ to achieve rapid dose falloff.

Prior to this work, SIMT treatments at our center were manually planned in the Varian Eclipse treatment planning system (TPS) using the Progressive Resolution Optimizer (PRO). A version upgrade from Eclipse version 15.6 to 18.1 required transition to the Photon Optimizer (as the PRO algorithm was removed from the TPS), thus we were required to implement an optimization approach using this algorithm. Varian's HyperArc was explored, but despite dosimetric benefits,^19^ we found the solution impractical due to its restrictive requirement of a specific immobilization setup, which imposed both cost and workflow challenges. Additionally, we identified areas where substantial planner time could be saved by automating parts of the process. Consequently, we opted to develop our own in‐house optimization tool that would not require specific software licenses nor be locked to a specific patient immobilization system.

In this study we present the design and validation of our in‐house SIMT planning tool, written using the Eclipse Scripting Application Programming Interface (ESAPI). Other authors have presented TPS scripts for SIMT planning. Kuo *et al.* described an ESAPI tool to automatically select the isocenter position and optimize collimator angles.^15^ Barrett *et al.* developed a tool to automatically generate optimization structures and load optimization objectives.^18^ A standalone program for full optimization of SIMT plans, which achieved similar plan quality to HyperArc, was presented by Mann *et al.* ^16^, ^17^ Our solution differs from the work of Kuo *et al.* and Mann *et al.* in that by design it does not perform any beam geometry optimization, instead leaving this to the expertise of the treatment planner. Like the tool presented by Barrett *et al.*, we automatically generate optimization structures and assign objectives. However, we also automated the optimization and dose calculation steps in our solution. Overall, the tool presented here automates more of the workflow than the tool described by Barrett *et al.*, but less than that of Mann *et al.* It also includes additional DVH convergence logic to satisfy our institutional requirements. In future work, it would be possible to incorporate additional automation such as beam geometry optimization, but this was not in the scope of the present tool. In our work, we applied the “ask for it” (AFI) technique described by Desai *et al.*,^3^ but other optimization schemes could also be used.

The outcome of this work was an Eclipse plug‐in script for SIMT plan optimization that both improved dosimetric plan quality as measured by dose conformality and falloff indices as well as saved planning time, as measured by a retrospective timing study. This software tool is now the standard method for planning cranial stereotactic cases at our institution.

## 2 MATERIALS AND METHODS

### 2.1 Institutional SIMT planning and treatment practices

In our institution, SIMT cases are treated with a range of 1, 3 and 5 fraction regimens, with 27 Gy in 3 fractions being the most common prescription. All SIMT patients are immobilized using CQ Medical equipment (CQ Medical, Avondale, PA): a Type‐S overlay (20CFHNSUB7) with Silverman type A or B headrests (MTSILVER2A or MTSILVER2B) and a 3.2 mm thermoplastic head only mask (RT‐1889KYSD). Patients are scanned for CT simulation on a Philips Brilliance Big Bore Oncology system with a reconstruction field of view of 35 cm. CT slice thickness is 1 mm and the reconstruction matrix size is 512 × 512. T1‐weighted MR imaging with gadolinium‐based contrast is rigidly registered to the planning CT scans for target delineation. Patients are treated on Varian Truebeam linear accelerators equipped with the Millennium120 multi‐leaf collimator (MLC). All SIMT treatments are delivered on machines equipped with the PerfectPitch 6‐DoF couch.

Treatment planning in our center is performed in the Varian Eclipse TPS. Our planning approach employs up to 5 arcs, with 2 of them being coplanar arcs at couch angle zero, and up to 3 non‐coplanar arcs at couch angles of 45, 315, and 90 degrees. Arc selection and collimator angle settings are at the discretion of treatment planners. Our institutional policy uses a 2 mm isotropic PTV margin around the GTV (or CTV if present, e.g. surgical cavities). Multi‐target stereotactic plans are optimized such that the PTV D_95%_ values for all targets lie within 1% of the target‐specific prescription dose. Additionally, the maximum dose within each PTV is constrained to 130% of the prescription dose. Typically, our plans would contain one, two and occasionally three different prescription dose levels for different targets, all with the same number of fractions. In practice, this approach requires multiple iterations of the VMAT *optimize‐calculate* loop, as the DVH curves presented in the optimizer interface are calculated with a simpler algorithm and at a different sampling grid than the final dose calculation.

### 2.2 Development of planning automation tool

#### 2.2.1 Optimization approach

In this work, the AFI optimization logic described by Desai et al.^3^ is implemented into an automatic optimization tool. This planning approach has been shown to achieve similar treatment plan quality as Varian's HyperArc solution.^14^

The AFI approach is a modified ring‐based optimization,^6^, ^7^, ^20^, ^21^ where the objective is to optimize the plan towards a “goal” value for R_50%_ (the ratio of the 50% isodose volume over the target volume) for each target. This is accomplished by constructing, for each PTV, an optimization shell with a margin size such that, in an “optimal” plan, 20% of the shell volume would be occupied by the 50% isodose line. A global outer shell (body contour minus PTVs and target‐specific shells) is also used to further minimize low dose spillage. Optimization objectives are then applied to both the target volumes and the optimization shells based on this goal. For this study, we maintained the ideal R_50%_ estimate used in the initial work by Desai *et al.*,^3^ which was originally derived empirically from the results of Ballangrud *et al.* ^4^ The calculations for appropriate shell margins and optimization objectives are described in Appendix A.

While the work of Desai et al.^3^ was originally proposed as a “single iteration” optimization strategy, our institutional policy requires alignment of the DVH curves to within 1% at D_95%_, often requiring several iterations of the optimize‐calculate loop. The auto‐optimization tool was designed to perform an optimization loop followed by a full dose calculation. Objectives are adjusted and the process is repeated until all target volumes fall within a user‐specified alignment threshold at the D_95%_ level, as described in Section 2.2.2.

#### 2.2.2 Software design

Figure 1 shows a flowchart of the automation tool's operation. The software is run as a binary plug‐in within the Eclipse TPS. On execution, the plug‐in initializes its user interface (UI) and populates the list of target volumes, as well as any previously defined optimization objectives. The tool does not delete existing objectives so that it can be run successively if desired. Additionally, in cases where a target volume is near a critical OAR, the planner can first open the standard Eclipse optimization window and add optimization objectives as needed. Similarly, if a monitor unit (MU) objective is desired, this must also be added in the standard Eclipse optimization dialog, as the scripting API does not provide access to MU objectives in the current version of Eclipse (18.1).

**FIGURE 1:**
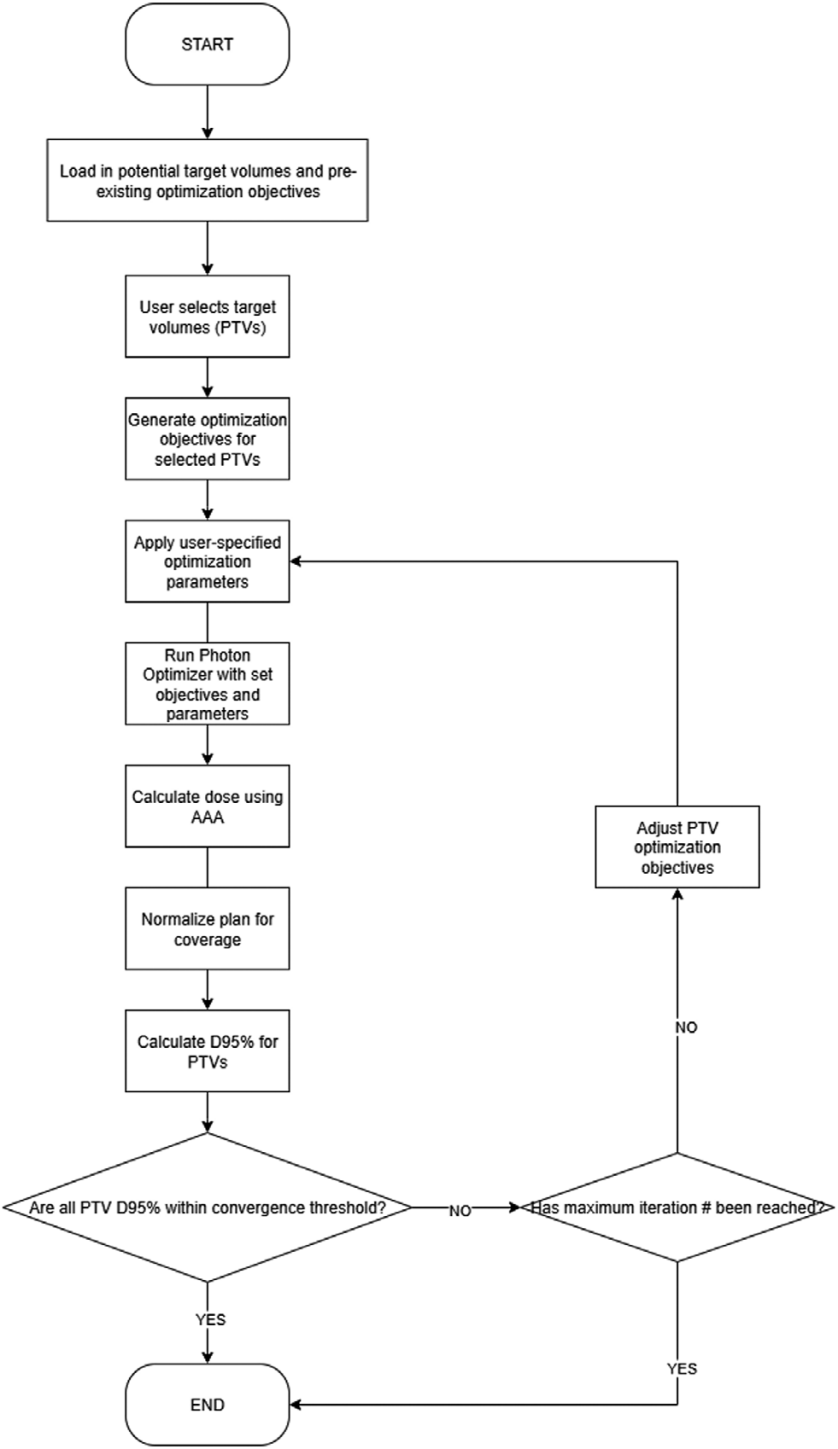
Flowchart showing operation of the automated optimization software tool. User‐specified parameters include the convergence threshold, maximum number of iterations, and SBRT NTO priority. These can be changed mid‐optimization.

PTVs and dose prescriptions are identified from the structure ID, as we enforce certain structure nomenclature rules in our practice. Target doses can be modified by the user within the UI prior to initiating the optimization. Individual targets can have unique dose prescriptions (provided all targets are prescribed to the same number of fractions). The user then selects which target volumes to include in the optimization, at which point the tool automatically generates appropriate objectives for the targets. Next, the user prompts the software to generate the optimization structures. The tool calculates the appropriate margin size for each PTV and generates the optimization shells. Any portions of PTVs present within optimization shells are cropped out of the shell volumes used prior to optimization. Appropriate optimization objectives for the shells are also calculated and applied at this stage. Once the user starts the automated optimization process, the program invokes the TPS optimization function, followed by the TPS final dose calculation function. After dose calculation, the plan is normalized for coverage such that 100% of prescribed dose covers 95% volume of one of the (highest prescription dose) targets in the plan. This target is maintained as the normalization target for all subsequent iterations. Relative differences between the rest of the PTV D_95%_ values and their prescription doses are then calculated. The dose values on the optimization objectives for each PTV are adjusted by the same relative magnitude and opposite sign as these differences, and the “optimize—calculate” process is performed again, followed by normalization. This process is repeated until the D_95%_ for each target is equal to the prescription dose within a user‐defined percentage range (the “convergence threshold”) or the user‐specified maximum number of iterations has been reached.

Figure 2 shows a screenshot of the tool's UI. Target volumes, prescribed doses, optimization objectives, convergence threshold, and maximum number of iterations can all be modified by the user. The SBRT normal tissue objective can also be enabled (but is not used in this work) by setting a priority greater than zero. Target‐specific optimization shells and objectives are generated when prompted by the user clicking the “Generate Optimization Shells” button. Modifications to objectives and parameters made mid‐optimization are applied to the next iteration of the optimization loop. DVH curves (reflecting the latest iteration's AAA dose calculation) for the target volumes are displayed on an interactive plot that can be zoomed in and out and read out using a mouseover data cursor.

**FIGURE 2:**
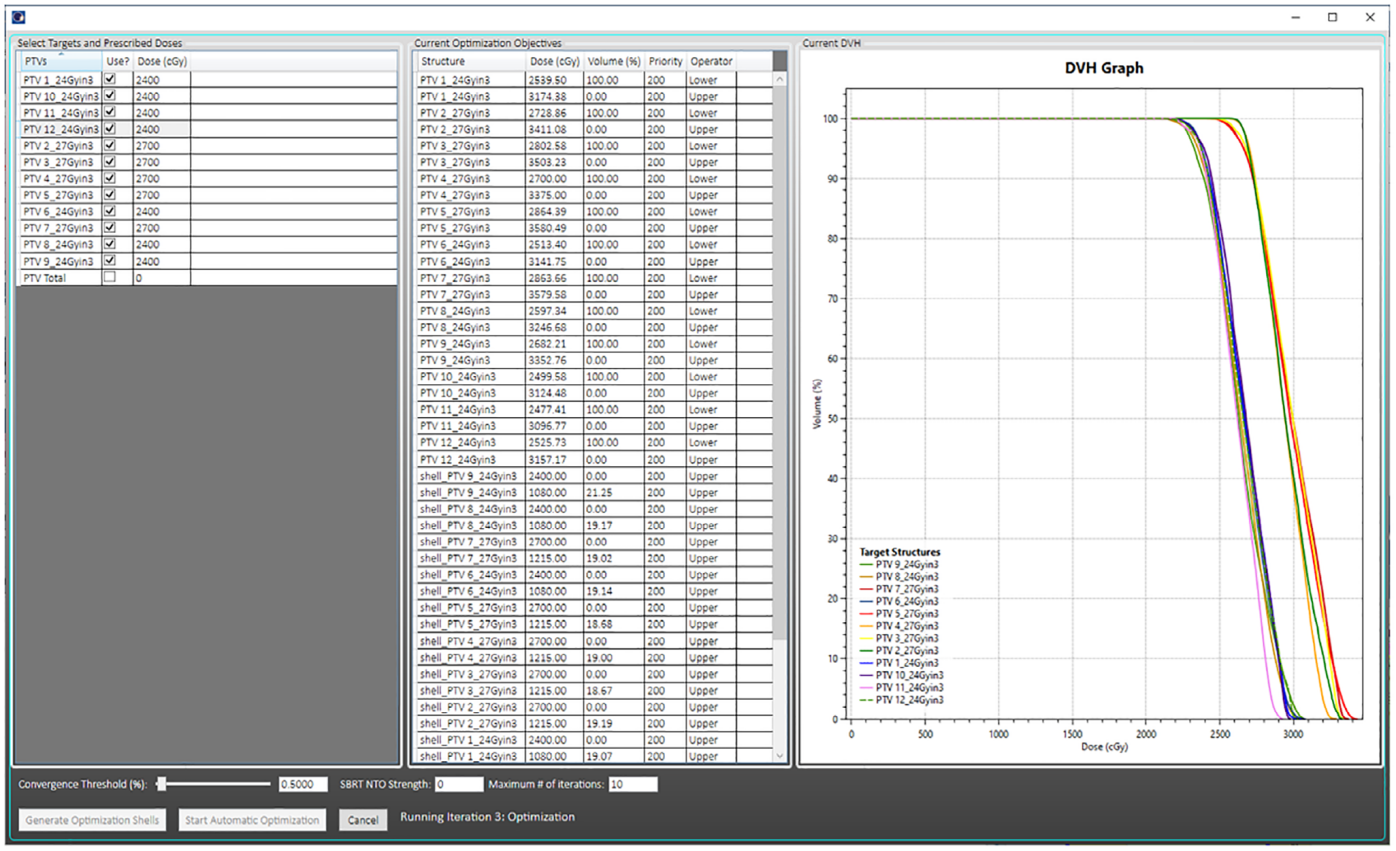
Automatic optimization tool user interface (UI) showing interactive target volume selection, optimization objectives, optimization parameters, and DVH curve visualization. The UI updates on each iteration (after dose calculation) to reflect current DVH curves and optimization objectives. Objectives and parameters can be modified mid‐execution by the user.

### 2.3 Retrospective planning study

A retrospective planning study was performed to evaluate the dosimetric performance of the AFI‐based tool against our previous clinical practice.

#### 2.3.1 Case selection

Twenty previously treated multi‐target cranial metastases plans, containing a total of 115 unique PTVs, were used for this study. These cases had previously been anonymized for education purposes and represented a randomly selected patient cohort. All cases were treated with 6MV beams and planned with our prior clinical optimization approach. Table 1 shows the number and size range of targets, prescription doses, and beam geometry used for treatment of each case. Some cases had target structures located close to critical OARs, where prescription doses for such targets are reduced to respect policy OAR tolerance doses. In this study, most cases were treated with a 3 fraction regimen with the majority of PTVs prescribed 27 Gy in 3 fractions, with some exceptions due to PTV size, OAR proximity, or previous treatment overlap. Different dose and fractionation regimens do not impact the functionality of the software tool. Clinically selected prescriptions for all target volumes were maintained for the retrospective planning study.

**TABLE 1:** Summary of cases used for retrospective planning study.

Case #	Number of PTVs	PTV Volume Range [cc]	Prescription Dose(s)	# of Arcs	Couch Angles for Arcs (°)
1	2	0.55–0.65	27 Gy/3Fx	2	0, 0
2	10	0.18–0.45	21 Gy/3Fx	5	0, 0, 45, 90, 315
3	6	0.46–1.8	24 Gy/3Fx (1 target)	5	0, 0, 45, 90, 315
			27 Gy/3Fx (5 targets)		
4	6	0.21–1.5	27 Gy/3Fx	4	0, 0, 45, 90
5	4	0.37–0.90	18 Gy/3Fx (1 target)	3	0, 0, 45
			27 Gy/3Fx (3 targets)		
6	3	0.17–12.1	18 Gy/3Fx (1 target)	3	0, 45,90
			27 Gy/3Fx (2 targets)		
7	2	0.24–0.60	27 Gy/3Fx	2	0, 0
8	4	0.91–5.1	18 Gy/3Fx (1 target)	3	0, 0, 45
			27 Gy/3Fx (3 targets)		
9	4	0.49–1.05	27 Gy/3Fx	3	0, 0, 45
10	5	0.27–1.7	27 Gy/3Fx	5	0, 0, 45, 90, 315
11	8	0.33–41.9	25 Gy/5Fx	5	0, 0, 45, 90, 315
12	7	0.25–92.24	35 Gy/5Fx	5	0, 0, 45, 90, 315
13	2	0.23–0.62	27 Gy/3Fx	2	0, 0
14	4	0.80–28.7	21 Gy/3Fx (1 target)	3	0, 0, 315
			27 Gy/3Fx (3 targets)		
15	12	0.32–2.53	24 Gy/3Fx (7 targets)	5	0, 0, 45, 90, 315
			27 Gy/3Fx (5 targets)		
16	6	0.19–2.03	27 Gy/3Fx	4	0, 0, 45, 315
17	7	0.53–63.42	32.5 Gy/5Fx	5	0, 0, 45, 90, 315
18	2	0.49–1.05	24 Gy/3Fx	2	0, 90
19	7	0.37–3.86	24 Gy/3Fx (2 targets)	5	0, 0, 45, 90, 315
			27 Gy/3Fx (5 targets)		
20	15	0.17–1.55	24 Gy/3Fx (2 targets)	5	0, 0, 45, 90, 315
			27 Gy/3Fx (13 targets)		

#### 2.3.2 Retrospective planning

Retrospective plans were generated using the automatic optimization tool. For fair comparison, the original beam geometry used in the clinical cases (Table 1) was maintained for retrospective plans. In all cases, the required convergence was set to 0.5% (meaning that the DVHs should align within 0.5% at D_95%_). No normal tissue objective was applied, as the optimization shells are used to enforce dose falloff instead. Initial optimization objectives on the target volumes and optimization shells are set as shown in Table 2. Note that we set an upper objective on targets at 125% of prescription dose, as our institutional policy aims to achieve less than 130% maximum dose in any given target. This limit could be released if higher central doses are allowed or desired. Critical OAR objectives were applied in cases 3, 5, 6, 8, and 12 for the optic chiasm and/or brainstem, with a dose value set 100 cGy below the maximum allowed point dose for that OAR and a priority of 500, as shown in Table 2. In all cases, a maximum of 10 optimization iterations was allowed.

**TABLE 2:** Initial optimization objective settings for targets and optimization shell structures. %*V_opt_* is calculated from the volumes of the PTV and PTV‐specific optimization shell, as well as the goal R_50%_ value, and typically has a value of approximately 20%. Where a critical OAR is deemed relevant, the OAR objective in the table is applied with our clinical OAR constraints. For the MU‐constrained optimization, an MU objective with a maximum MU of 2.5‐3.0x the highest prescribed daily dose (in cGy) was applied with a priority setting of 80.

Structure	Objective Type	Dose Value (% of prescribed)	Volume (%)	Priority
PTVs	Lower	100	100	200
	Upper	125	0	200
Optimization Shells	Upper	100	0	200
	Upper	45	*%V_opt_*	200
Global Outer Shell	Upper	45	0	200
OAR (if applicable)	Upper	[max allowed point dose – 100 cGy]	0	500

Two different automatic optimization scenarios were evaluated. First, plans were optimized with no MU objective specified in the optimizer (MU‐unconstrained). Second, plans were optimized with a monitor unit objective set at 2.5 ‐ 3.0 times the daily dose (in cGy) with a priority weighting of 80 (MU‐constrained). The value of 2.5 times the daily dose was selected based on internal experience and consultation with other centers on their SRS optimization strategy. For cases with larger PTV volumes (i.e. 20+ cc), many targets (10+), or targets in close proximity to critical OARs, it was sometimes necessary to release this to a factor of 3.0 to achieve an acceptable plan. In this study, this was necessary for cases 11, 12, 15 and 20 in Table 1. A “most optimal” setting for the MU objective was not studied further in this work.

It should be noted that in the current version of the tool, to set an MU objective or an OAR objective, planners are required to open the regular Photon Optimizer dialog window (i.e. begin optimization), specify and apply the objectives, and then close the window. This is partially due to a limitation of the current version of the Eclipse Scripting API, which does not expose the MU objective.

#### 2.3.3 Optimization and dose calculation algorithms and calculation hardware

Retrospective optimization was performed using the Photon Optimizer (PO), version 18.1.1 on a GPU‐equipped FAS server, with the GPU optimization option selected. The PO Convergence Mode option was enabled, and optimization was performed at the “normal” 2.5 mm grid spacing. An intermediate dose calculation was enabled for the first iteration of the process. Dose calculation was performed using the Analytical Anisotropic Algorithm (AAA), version 18.1.1. The calculation grid spacing was set to 0.2 cm (2 mm), as per our institutional policy.

Optimization and dose calculation jobs were sent to a Distributed Calculation Framework (DCF) consisting of 12 framework agent servers (FAS) (Dell PowerEdge R760), which each had 40 CPU cores (2x Intel Xeon Silver 4416+) and 256 GB of RAM. Additionally, five FAS were equipped with GPUs (4x NVIDIA L4 Tensor Core, 24GB VRAM). Optimization was exclusively performed using the GPU‐based PO algorithm. Dose calculation was distributed over up to 12 agents. For all calculations in this study, no other jobs were in progress on the DCF.

#### 2.3.4 Plan evaluation

The automatically optimized plans were qualitatively and quantitatively compared against the previously treated clinical plans.

##### Qualitative evaluation

Previous clinical plans were already evaluated as part of clinical practice. Newly generated automated plans were reviewed by a medical physicist for overall clinical appropriateness. All plans were evaluated according to the same institutional standards applied for routine plan quality assurance (QA). Broadly, this included assessment of the dose distribution, including target conformality and sparing of organs at risk (OARs), as well as visual inspection of multileaf collimator (MLC) motion to ensure leaf movements appeared reasonable. While this evaluation was unblinded and mostly qualitative, it was still used to verify that the auto‐optimization tool generated plans that met or exceeded institutional requirements.

##### Quantitative evaluation

Quantitative analyses were performed to better compare automated plans against the previously treated clinical plans. All statistical analyses were performed using R version 4.5.1.^22^

The following global metrics were calculated for all plans: Volume receiving 50% of max prescribed dose (V_50%_), Volume receiving 30% of prescribed dose (V_30%_), number of monitor units used in plan (MU), percentage of total monitor units assigned to couch nonzero‐degree arcs (%NZ), average aperture size per control point (AA),^23^ and Modulation Complexity Score (MCS).^23^, ^24^ V_50%_ and V_30%_ are used to measure the overall moderate to low “dose‐wash” present in the plan. These relative values were chosen over more commonly published absolute metrics (e.g. V12Gy), as prescriptions for the plans in the study were not all identical. The number of monitor units, AA, and MCS are used to evaluate plan complexity, where more complex plans in general have higher MUs, smaller AA, and smaller MCS than less complex plans. The distribution of monitor units between couch zero‐degree and couch nonzero‐degree arcs was measured to help explain differences in V50% and V30% between automated and previously generated clinical plans. All global plan metrics were compared statistically using a one‐way, repeated measures ANOVA. Post‐hoc pairwise comparisons were performed using the paired Wilcoxon test with Bonferroni correction applied.

In addition to global plan metrics, PTV‐specific metrics were calculated for all target volumes in all plans. The Conformity index (CI) and R_50%_ metrics, as applied by Desai *et al.*,^14^ were calculated for each target in each plan by using the V_100%_ and V_50%_ values for each PTV and corresponding optimization shell, respectively. The Paddick CI^25^ and Gradient Index (GI)^26^ were calculated for each target using the same shell‐based approach. PTV‐specific values were necessary because it is not possible or useful to calculate an overall CI, GI, or R_50%_ for a multi‐target plan. Differences in PTV‐specific metrics between optimization approaches were tested using one‐way, repeated measures ANOVA and paired Wilcoxon (with Bonferroni correction) post‐hoc pairwise comparisons.

##### Plan deliverability and patient specific quality assurance

All plans were assessed for clinical deliverability using Portal Dosimetry (Varian Medical Systems). Portal Dose prediction images were calculated using the Portal Dose Image Prediction (PDIP) algorithm, version 18.1.1. The PDIP algorithm, as well as the MV imagers in our center, are configured using the preconfigured portal dosimetry package available from Varian.^27^, ^28^ Portal dosimetry analysis was performed using gamma analysis with 3%/2 mm dose difference/distance to agreement thresholds (Global gamma evaluation, within the MLC CIAO + 1.0 cm margin, with threshold of 5.0%). This is more stringent than our standard institutional practice of 3%/3 mm. A minimum pass rate of 95% of pixels having gamma < 1.0 was considered a pass.

### 2.4 Software planning efficiency evaluation

To quantify the time savings achievable with the tool, an experienced treatment planner performed manual re‐optimization of the first 10 cases in Table 1 and recorded the time required to obtain a clinically acceptable plan, based on our institutional criteria. Times were measured from the start of the first optimization iteration to the point at which an acceptable plan was achieved. Therefore, this timing study only examined the optimization time and did not account for any contouring time savings.

## 3 RESULTS

### 3.1 Qualitative evaluation of automated plans

Figure 3 shows representative axial, coronal and sagittal views through one of the cases, with both the automated (MU‐constrained) plan and the previous clinical plan displayed side by side. The auto‐optimization tool generated plans that met all clinical DVH constraints and were deemed to be clinically appropriate by a medical physicist following our institutional policies.

**FIGURE 3:**
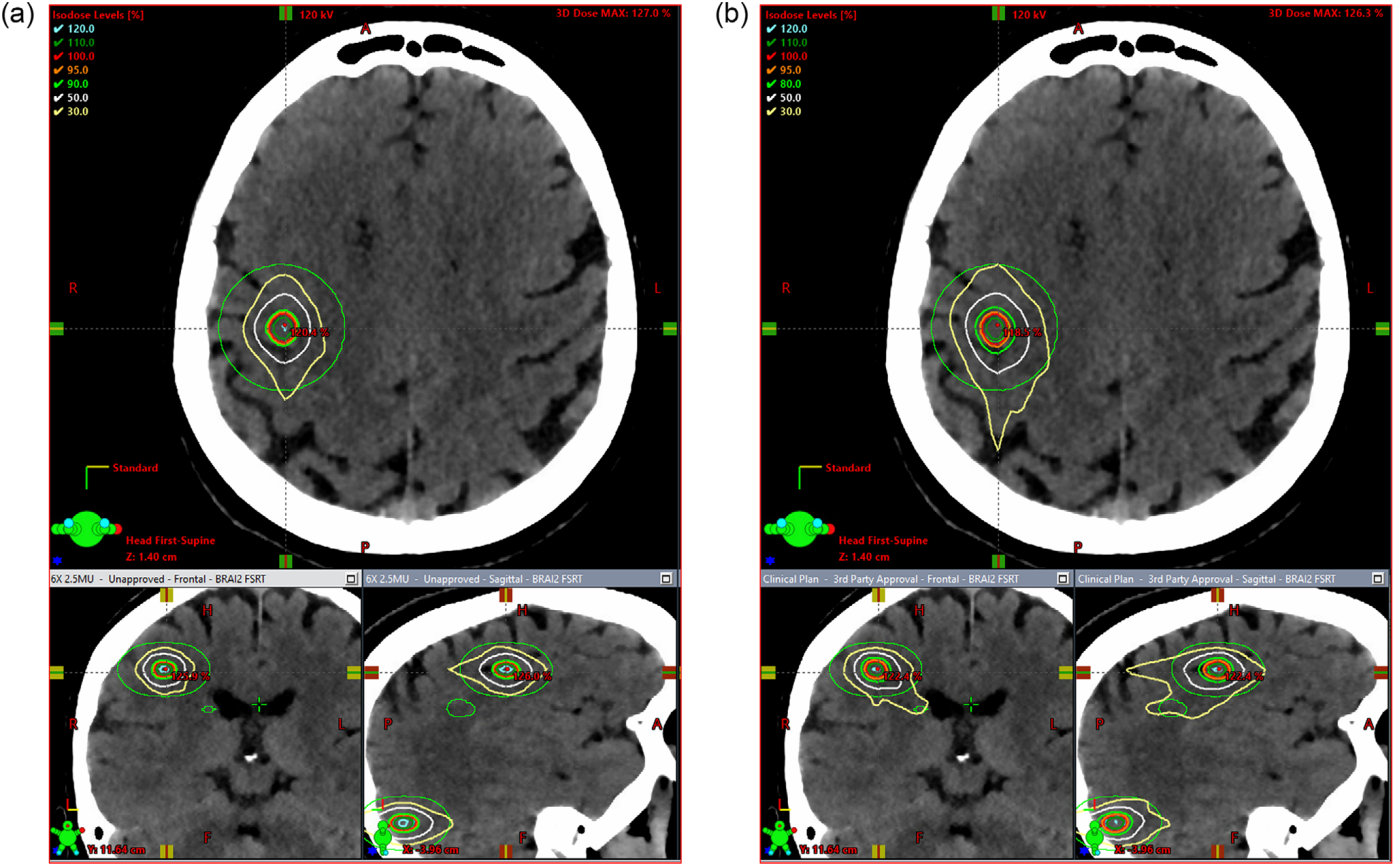
Axial, coronal, and sagittal slices through (a) automatically‐optimized (MU‐constrained) plan and (b) previously treated clinical plan for Case 10. Isodose levels of 30, 50, 95, 100, 110, and 120% of the prescription dose are displayed. The reduction in size of the 30% and 50% isodose lines in the retrospective plan can be clearly appreciated.

### 3.2 Quantitative comparison of automated and clinical plans

Table 3 presents the statistical comparisons of global plan metrics and PTV‐specific metrics between the original clinical plans and the retrospectively generated automated plans, with and without the application of the MU–constraining objective.

**TABLE 3:** Quantitative comparisons between previously delivered clinical plans and automated retrospective plans generated with and without MU‐constraining objectives applied. Adjusted *p*‐values for Wilcoxon pairwise comparisons after the ANOVA are also reported. PTV coverage statistics are not shown, as all PTV D_95%_ were within 1% (clinical plans) or 0.5% (retrospective automatic plans) of their prescription dose after optimization.

Parameter	Clinical plan		Automatic, MU‐unconstrained		Automatic, MU‐constrained		*p*‐values^*^		
Overall plan statistics									
	Mean	S.D.	Mean	S.D.	Mean	S.D.	Clin. vs. MU‐unc.	Clin. vs. MU‐con.	MU‐con. vs MU‐unc.
Total MU	2289	514	2859	652	2148	257	0.0001	0.66 *n.s*.	0.0001
%NZ^**^	31.5	12.0	42.2	11.3	43.7	10.1	0.003	0.003	0.107 *n.s*.
V_50%_ [cc]	72.8	97.2	63.4	88.1	68.9	97.7	3e‐5	0.002	0.035
V_30%_ [cc]	217.4	264.6	202.2	269.5	207.3	286.3	0.041	0.036	1.0 *n.s*.
AA [mm^2^]	729	514	550	361	831	615	0.003	0.364 *n.s*.	8e‐5
MCS	0.213	0.055	0.198	0.034	0.233	0.049	0.348 *n.s*.	0.261 *n.s*.	0.001

**Individual PTV statistics**									
	Mean	S.D.	Mean	S.D.	Mean	S.D.	Clin. vs. MU‐unc.	Clin. vs. MU‐con.	MU‐con. vs MU‐unc.
CI	1.25	0.55	1.09	0.13	1.11	0.15	0.0001	0.0001	0.384 *n.s*.
Paddick CI	0.79	0.15	0.84	0.08	0.83	0.09	0.0004	0.004	0.019
R_50%_	10.14	5.41	7.61	3.17	8.29	3.71	2e‐18	2e‐16	2e‐13
Paddick GI	8.06	3.24	6.82	2.26	7.25	2.53	3e‐11	6e‐7	2.5e‐14

* *p‐*values reported in triplet for pairwise comparisons between 1. Auto, MU‐unconstrained vs. Clinical, 2. Auto, MU‐constrained vs. Clinical, 3. Auto, MU‐constrained vs. Auto, MU‐unconstrained. Results not reaching statistical significance are indicated by *n.s*.

** %NZ denotes the fraction of total plan MUs assigned to the couch nonzero‐degree arcs.

Figures 4, 5, and 6 show plots of the target‐specific R_50%_, Paddick CI, and Paddick GI values obtained for all three optimization scenarios.

**FIGURE 4:**
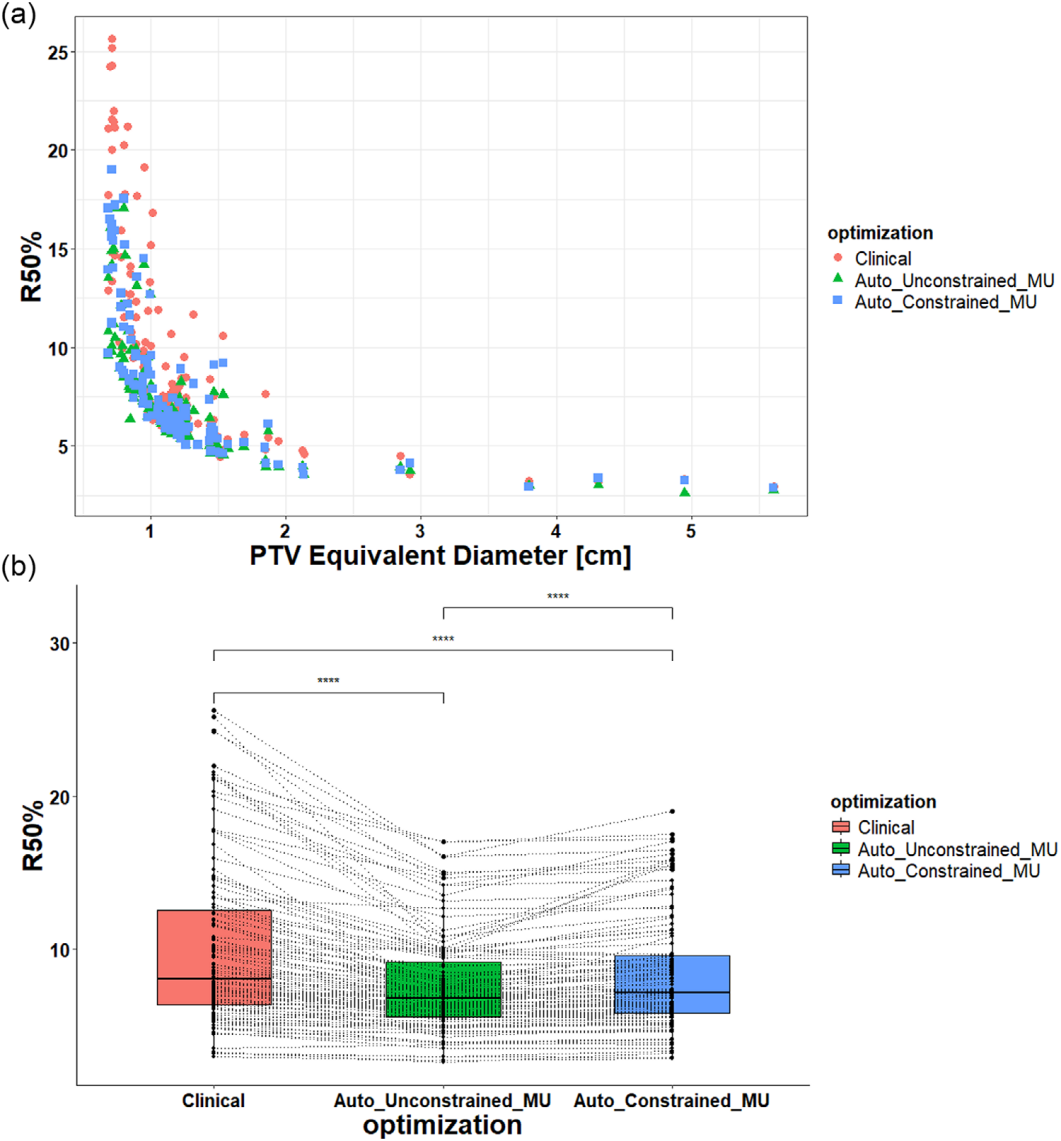
PTV‐specific R_50%_, displayed as a function of PTV equivalent diameter (a) and in paired boxplot form (b), for each optimization approach. In the boxplot, dotted lines connect results from each individual PTV across the 3 optimization scenarios.

**FIGURE 5:**
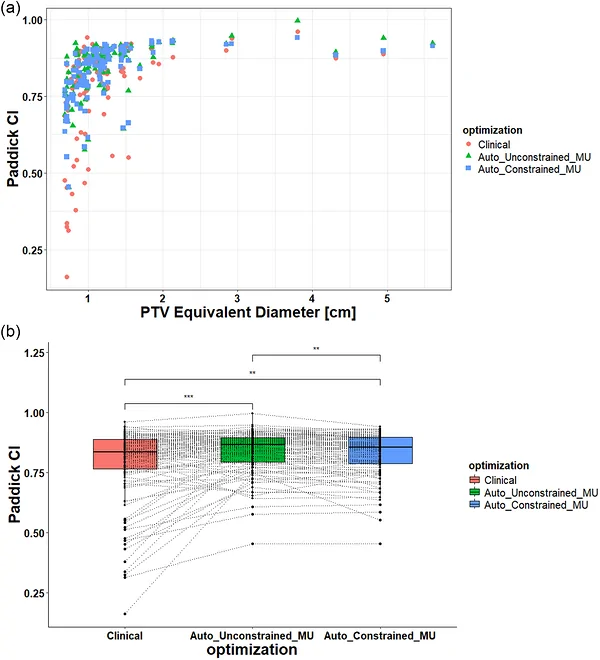
PTV‐specific Paddick CI, displayed as a function of PTV equivalent diameter (a) and in paired boxplot form (b), for each optimization approach. In the boxplot, dotted lines connect results from each individual PTV across the 3 optimization scenarios.

**FIGURE 6:**
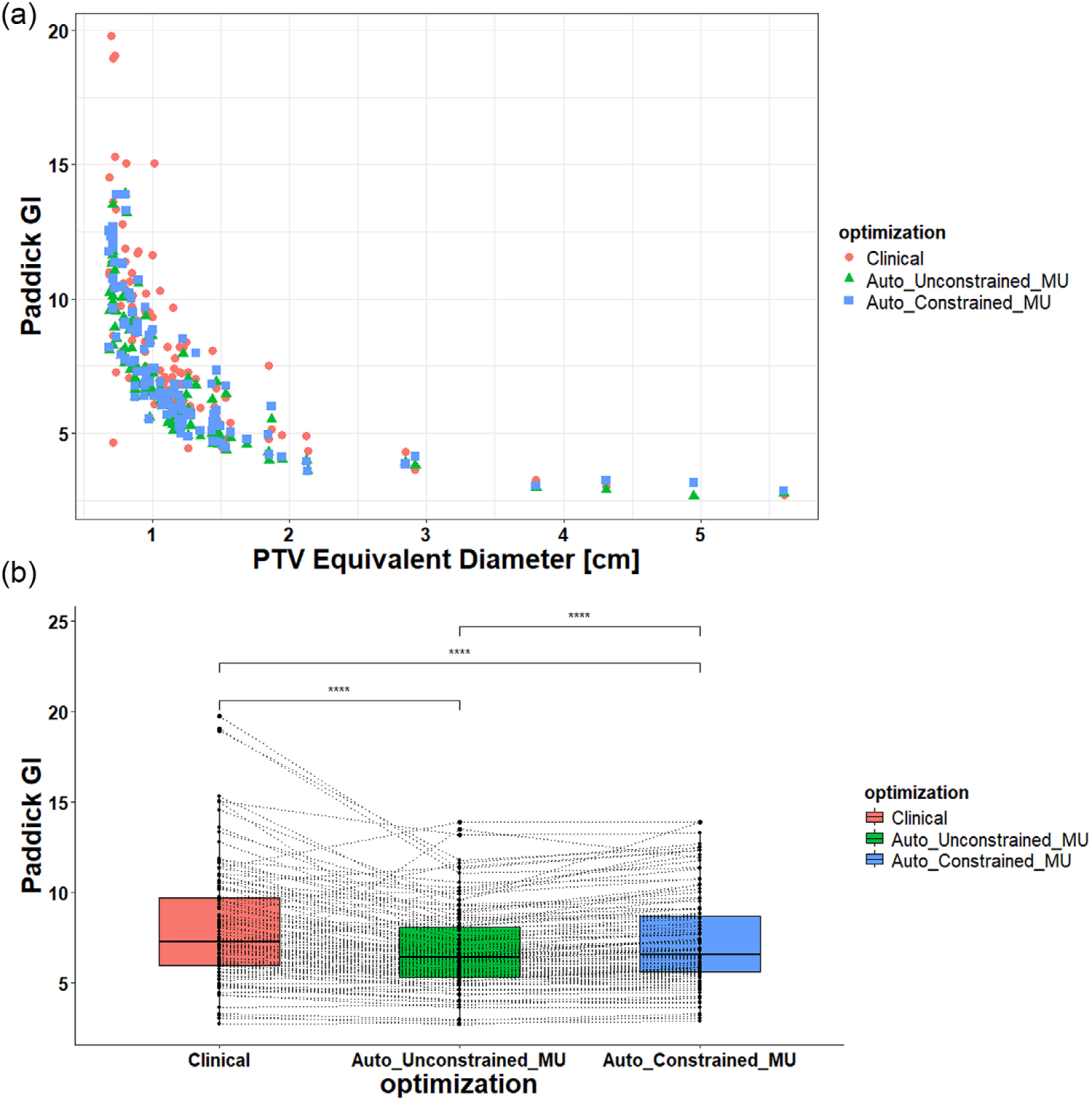
PTV‐specific Paddick GI, displayed as a function of PTV equivalent diameter (a) and in paired boxplot form (b), for each optimization approach. In the boxplot, dotted lines connect results from each individual PTV across the 3 optimization scenarios.

In both automated plan conditions, statistically significant decreases in both V_50%_ and V_30%_ were observed compared to clinical plans, with reductions of approximately 5–15% and 5–7% respectively, depending on the use of the MU constraint. Differences between the two automated optimization techniques were statistically significant for V_50%_ but not for V_30%_ in this study.

The automatically optimized plans demonstrated a statistically significant difference in the weighting of couch nonzero‐degree arcs. On average, these plans allocated approximately 43% of the total monitor units (MU) to couch nonzero‐degree arcs, compared with approximately 32% in the original clinical plans. No significant differences in the weighting of non‐zero degree arcs were observed between the two automated planning approaches (MU‐unconstrained vs. MU‐constrained).

When a monitor unit limiting objective is not applied, there is a statistically significant increase in the number of monitor units used to generate the plan, with a mean increase of 25% compared to the clinical plan. A mean reduction in the average aperture size of approximately 25% was observed compared to the clinical plan. There was no significant change to the modulation complexity score.

When a monitor unit limiting objective is applied, there is no statistically significant change in the number of monitor units used to deliver a plan compared to the clinical plan. The difference in the weighting of nonzero‐degree couch arcs remains significant. With the MU limiting objective, there is no statistically significant change in the AA or MCS compared to clinical plans, however there are statistically significant increases in these parameters between MU‐constrained and MU‐unconstrained automated plans.

In this study, we observed statistically significant changes in CI, Paddick CI, R_50%_, and Paddick GI when moving from the clinical plans to either of the automatic optimization approaches. Both types of automated plans displayed a lower CI, Paddick GI, and R_50%_ compared to clinical plans, and a corresponding increase in Paddick CI. When moving from MU‐unconstrained to MU‐constrained automatic optimization, there was a small increase in CI, Paddick GI, and R_50%_ values, with a corresponding decrease in Paddick CI.

### 3.3 Plan deliverability

All automatically generated plans were successfully delivered as portal dosimetry plans. Analysis of all EPID images with gamma analysis at dose difference / distance to agreement criteria of 3%/2 mm and 3%/1 mm passed with a minimum threshold of passing pixels of 95%. The average pixel pass rate for clinical plans was 98.4% at 3%/2 mm and 96.6% at 3%/1 mm, compared to 98.8% and 97.2% for MU‐unconstrained plans, and 98.4% and 96.5% for MU‐constrained plans. Differences in pass rates between optimization types were not statistically significant.

### 3.4 Software performance

For the 10 cases included in the software performance evaluation, Table 4 summarizes the optimization time required for manual planning, as well as for the software tool to complete optimization of each case in both automated planning scenarios.

**TABLE 4:** Overall optimization time and number of iterations required for convergence for the manual reoptimization, as well as the automated plans (MU‐unconstrained and MU‐constrained). The average optimization times were 1285 seconds for manual planning, 310 seconds for unconstrained automated plans, and 385 seconds for constrained automated plans. For manual plans, an average of 3.8 iterations of the “optimize—Calculate” loop was required. For automated plans, the average number of iterations required for MU‐unconstrained optimization was 3.6 vs. 4.2 for MU‐constrained optimization.

			Manual Optimization		Automated Optimization			
					MU‐unconstrained		MU‐constrained	
Case #	# PTVs	PTV Volume Range [cc]	Time (s)	# Iter	Time (s)	# Iter	Time (s)	# Iter
1	2	0.55‐0.65	222	1	184	3	148	2
2	10	0.18‐0.45	2955	7	684	6	619	5
3	6	0.46‐1.8	937	2	326	2	223	3
4	6	0.21 ‐ 1.5	1696	4	220	3	597	6
5	4	0.37‐0.90	1860	7	482	5	523	7
6	3	0.17 ‐ 12.1	1657	6	247	5	285	4
7	2	0.24‐0.60	330	1	177	2	190	2
8	4	0.91 ‐ 5.1	963	3	221	4	374	4
9	4	0.49‐1.05	673	2	123	2	376	4
10	5	0.27‐1.7	1560	5	433	4	510	5

## 4 DISCUSSION

### 4.1 Plan quality

Plans generated using the auto‐optimization tool were of equivalent or superior quality compared with the previously treated clinical plans. All automated plans met established clinical DVH constraints and were deemed clinically acceptable under institutional policy by a medical physicist. No atypical dose distributions or poorly conforming plans were identified on visual inspection. Visual evaluation of the planned MLC motion did not reveal any irregular or concerning modulation patterns. Qualitatively, the automatically generated plans demonstrated a more spherical isodose distribution surrounding individual targets. Although these assessments are inherently subjective, they reflect a critical component of the clinical treatment planning quality assurance process. Quantitatively, the automated approach achieved a statistically significant reduction in V_50%_ and V_30%_ compared to previous clinical plans, with approximate decreases of 10%–12% on average.

On a per‐target basis, CI values were reduced from an average value of 1.25 to 1.10, indicating that the software resulted in less high‐dose spillage around targets than previous clinical plans. Correspondingly, the Paddick CI values increased from an average of 0.79 to approximately 0.83–0.84. The standard deviation in the CI and Paddick CI values was also considerably reduced, suggesting a more consistent planning approach than manual planning. The Paddick CI values reported in this study for the automated tool are comparable to those found in other publications relating to SIMT planning using HyperArc.^5^, ^12^, ^14^ Additionally, they are similar to those reported by Mann *et al.* when applying their standalone ESAPI tool.^16^, ^17^ It should be noted that our work was performed using a Millennium120 MLC, while many of the previous studies made use of an HD120 MLC, which has 2.5 mm central leaf widths as compared to 5 mm. These results indicate that our implementation produces results comparable to other good‐performing solutions in terms of high dose conformality.

In terms of dose falloff metrics, the R_50%_ value decreased by 20–25% on average compared to the previous clinical plans, with some dependence on whether MU limiting objectives were applied in optimization. The R_50%_ values reported in this study are higher than those presented in the initial work by Desai *et al.* ^3^ This discrepancy is partly related to the use of Millennium MLC in our study compared to the HD‐MLC used in their work, which does result in increased low to intermediate isodose volume sizes in SIMT planning.^29^ Differences are also partly attributable to the close spatial proximity of some targets in the present cohort, resulting in overlap of optimization shells between adjacent targets. Consequently, the volume of a given shell receiving 50% of the prescription dose may include contributions from multiple target volumes. As such, R_50%_ represents a limited metric in multi‐target scenarios; however, it remains a useful parameter for comparative evaluation of different optimization strategies applied to the same case. Similarly, the Paddick GI values decreased by approximately 15% in the MU‐unconstrained optimization and by 10% in the MU‐constrained optimization compared to the previous clinical plans. Our tool obtained mean Paddick GI values of approximately 7.0, using the 100% isodose as our prescription isodose volume. This was larger than the Paddick GI values reported by Popple *et al.* using HyperArc (5.42), however almost 60% of the cases in their study had a single target^5^ and their entire cohort was planned using a linac equipped with an HD‐MLC. Mann *et al.* used a GI defined as the ratio of the equivalent sphere diameters of the 50% and prescription isodose volumes. They obtained median GI values of 1.85 using their automatic optimization software and 1.68 using HyperArc,^17^ also using an HD‐MLC. Computing GI values based on their definition with our data, we obtained median values of 1.86 and 1.87 with MU‐unconstrained and MU‐constrained optimization using our tool, indicating that we achieved relatively similar falloff to their work, despite the MLC difference.

Improvements in the target‐specific metrics compared to our clinical plans were generally consistent across the range of PTV sizes present in our study, as can be observed in Figures 4a, 5a, and 6a. It should be noted that we strive to achieve a maximum target dose criterion in our center, which generally results in a slight increase in dose falloff metrics. Relaxing this criterion would likely improve our R50% and Paddick GI metrics.

There were no statistically significant changes in complexity metrics between the clinical plans and either the MU‐unconstrained or MU‐constrained automated plans, however the constrained plans were statistically less complex than the unconstrained plans and required significantly fewer MU to deliver. The slight increase in R_50%_ between MU‐unconstrained and MU‐constrained optimization was deemed acceptable to realize reduced beam on time and to assuage any potential concerns over delivery accuracy (as the less complex plans with larger MLC apertures will be subject to less uncertainty associated with MLC modeling^30^). In fact, the MU‐constrained automated plans are of similar or lower complexity than those generated by the previous clinical practice. It should be noted that we did not have concerns over the delivery accuracy of any of the plans generated in this study, and all plans passed patient‐specific QA using Portal Dosimetry.

It should be noted that the previous clinical plans were generated using the PRO algorithm, while the automated plans for this study used the PO algorithm. This introduces some uncertainty in the dosimetric comparison between plans. However, subsequent PO‐based manual optimization of select plans in our study using the original clinical optimization strategy resulted in very similar plans as the previous PRO optimizations. This means that most of the differences between the optimization results achieved with our software tool and those previously obtained clinically are related to the change in optimization philosophy from a single global ring structure to a set of target‐specific shells, rather than intrinsic differences between algorithms.

At our center, SIMT plans are optimized with the goal of achieving a maximum dose value of 130% of prescribed dose for each target. This practice was maintained in the design of our automation tool; however we note that falloff metrics may be further improved by relaxing this criteria. Additionally, we calculate dose using a 0.2 cm grid spacing, while many other centers use 0.1 cm spacing for stereotactic plans. In our center, we have observed that dose calculated with finer spacing does agree better with very fine resolution dosimetry measurements; however, we have not changed our calculation settings institutionally because this would have required a change in plan normalization to maintain consistency in actual delivered doses. We did not wish to introduce any clinical practice changes, therefore the existing maximum dose and calculation grid spacing was maintained for this work.

### 4.2 Software performance

The mean optimization time across the 10 cases included in the speed evaluation portion of this study was 310 seconds (5.2 min) without MU constraints and 385 seconds (6.3 min) with MU constraints. For comparison, the mean optimization time for manual planning was 1285 seconds (21.4 min), which was based on a single experienced planner. Cases 1, 7 and 9 were optimized very rapidly by the tool because they had few targets and only two arcs utilized (in case 1, only two 180 degree arcs were used). On the high end, even highly complex cases (e.g. Case #2, 10 targets; Case #6, large range in target sizes and target within critical OAR) were optimized in approximately 10 min or less. This evaluation did not consider the additional time savings achieved by automatically generating the optimization shells. Although optimization without monitor unit (MU) constraints was marginally faster, the difference was small relative to the overall time savings. Accordingly, the application of MU constraints is preferred to limit plan modulation complexity, as discussed in Section 4.1.

### 4.3 Limitations and future work

There are some limitations to the work presented in this study. First, our work was done entirely using the Millennium120 MLC, while much of the previously published work makes use of HD‐MLCs. This results in some degradation of the achievable plan quality for very small target volumes^29^ and makes comparisons with other work slightly challenging. The tool implemented in this study would be capable of handling HD‐MLCs, but we do not currently have any units equipped with them. Additionally, the goal R_50%_ value used in the current version of the software may be insufficiently ambitious to obtain the best possible falloff for a given case. Use of the R_50%,Analytic_ value presented by Desai *et al.* ^31^ to generate the optimization objectives will be examined in a future version, as this value will be consistently lower than what was used in the present work. One limitation of the retrospective planning study in this work is that we did not have a singular radiation oncologist review all plans to directly provide clinical opinion on plan quality. However, all previous clinical plans were initially approved as clinically acceptable by radiation oncologists, and that in virtually all cases, the dose falloff metrics were improved in the retrospectively planned cases. Additionally, following the clinical implementation of this tool, almost all our SIMT cases have been planned using it at our center with no concerns or comments raised by the physicians. Finally, our work was limited to the Eclipse TPS and does not apply to other planning systems.

Several enhancements to the software could be considered. First, the ability to create and remove optimization objectives for arbitrary structures from within the UI could improve usability, eliminating the need for the user to predefine non‐target objectives in the TPS optimization workspace. This functionality is supported by the existing API and could be incorporated in future development. Second, the ability to modify the monitor unit objective from within the tool's UI is desirable as it would enable the full optimization process to occur without entering the regular optimizer window. However, in the current version (18.1) of ESAPI, the MU objective is not exposed to API calls and cannot be modified programmatically. Implementation of this feature would require support in a future API release. Additional future improvements include toggleable DVH curves for both targets and organs at risk (OARs), as well as expansion of user‐adjustable optimization parameters (e.g. allowing the user to more easily modify the maximum dose allowed in a plan and to select the plan normalization method).

It should be noted that the tool presented here does not perform beam geometry optimization, which has a substantial impact on plan quality. This means that our planning process continues to rely on the expertise of treatment planners. This does imply that less experienced treatment planners may not be able to use our tool to achieve the level of plan quality presented in this study. In this work, we did not wish to remove the influence of treatment planner skill by design. However, future versions could involve implementation of beam geometry optimization logic and restructuring the software as a standalone application operating independently of the TPS, with user interaction limited to the initiation of the optimization process, as was done by Mann *et al.* ^16^ Such a program could even be run as a background service that could be assigned a queue of plans to optimize, which would further improve efficiency in higher‐throughput clinical environments.

The tool presented in this work would be easy to implement in another existing SIMT program, as it was designed to be completely agnostic to parameters such as the choice of immobilization devices, beam energy, prescription doses, and dose calculation settings. Additionally, no software licenses beyond access to the Eclipse Scripting API were required. In terms of software development, the tool was completely built by a single medical physicist with computer programming experience, a resource that many clinics likely have access to should they wish to pursue similar efforts. Implementing the tool clinically at our center required minimal training, as it is simply run from the Eclipse script menu without requiring any standalone applications to be launched. Certain optimization goals and parameters were hardcoded for our center in this version, but in a wider distribution of the software this could be changed to allow more flexibility as described above.

## 5 CONCLUSION

The auto‐optimization tool presented in this work improves planning of SIMT stereotactic cranial treatments in our institution both in terms of plan quality (as measured by dose falloff around targets) and overall planning efficiency. Based on these findings, the software has been adopted as the default approach for cranial stereotactic treatment planning within our center.  Although the present study was limited to retrospective planning using 6 MV flattened photon beams, the tool works equally well in treatment planning with flattening filter free beams. While certain features are tailored to our institutional practices, such as the iterative approach used to align DVH curves, other components of the software, including the automated generation of optimization shell structures, should have broader appeal. Additionally, our solution does not require any specific software licenses beyond access to the Eclipse Scripting API, and is not locked to any specific immobilization device, which reduces costs. This is our center's first implementation of an in‐house plan automation script. Experience gained through this work is expected to facilitate the development of future scripting initiatives at our institution.

## AUTHOR CONTRIBUTIONS

KD designed, programmed, and tested the software tool, designed the retrospective planning study, performed the tool‐assisted retrospective planning, performed data analysis, and wrote and edited the manuscript. JE performed testing of the software tool, contributed to study design, performed manual planning for the retrospective planning study, and reviewed and edited the manuscript.

## CONFLICT OF INTEREST STATEMENT

The authors declare no conflicts of interest.

## ETHICS STATEMENT

This retrospective study was approved by the institution's ethics board.

## Data Availability

The plan quality and DVH metrics supporting the findings of this study are available upon reasonable request to the corresponding author.

## A.1 Calculation of optimization shell margins and optimization objective volume

The AFI approach presented by Desai *et al.* 3 is programmed into the software tool presented in this work. The derivation of the relationships between PTV volume and optimization shell margin is presented in their report. Here, we reproduce the final derived equations only, for convenience.

The equation for the margin M(in cm) for a given PTV with volume $V_{\mathrm{PTV}}$ (in cubic cm) is:

(A1)
$$M={\left(\frac{3}{4\pi }V_{\mathrm{PTV}}\right)}^{1/3}\;\times \left[{\left(5\times R50{\%}_{\mathrm{Goal}}-4\right)}^{\frac{1}{3}}-1\right]$$

Where $R50{\%}_{\mathrm{Goal}}$ is given by:

(A2)
$$R50\;{\%}_{\mathrm{Goal}}=4.8{\left(V_{\mathrm{PTV}}\right)}^{-0.2}$$

Equation (A2) was reported in the work by Desai *et al.* based on data on “optimal” plans presented by Ballangrud *et al.* ^4^

In addition to the margin size M, a relative volume of each shell that should receive 50% of the prescription dose for the PTV is calculated to inform the optimization objectives. The calculation of this volume for each shell, $\%V_{\mathrm{opt}}$, is calculated from:

(A3)
$$\%\;V_{\mathrm{opt}}=\frac{100\times \left(R50{\%}_{\mathrm{Goal}}-1\right)\times V_{\mathrm{PTV}}}{V_{\mathrm{shell}}}$$

Where $V_{\mathrm{shell}}$ is the volume of the optimization shell for the PTV.

## A.2 Calculation of PTV‐specific R_50%_, CI, Paddick CI, and Paddick GI

Calculation of R_50%_ and Conformity Index for a single target plan is straightforward, as both are simply ratios of a given isodose volume over the volume of the PTV, i.e.

(A4)
$$\;R_{50\%}=\frac{V_{50\%\;\mathrm{isodose}\;}}{V_{\mathrm{PTV}}}$$

(A5)
$$\mathrm{CI}\;=\frac{V_{100\%\;\mathrm{isodose}\;}}{V_{\mathrm{PTV}}}$$

Similarly, the Paddick Conformity Index and Paddick Gradient Index can be shown to be given by:

(A6)
$$\;CI_{\mathrm{Paddick}}=\frac{{\left(V_{100\%,\;\mathrm{PTV}\;}/\;V_{\mathrm{PTV}}\right)}^{2}}{\mathrm{CI}}$$

(A7)
$$\;GI_{\mathrm{Paddick}}=\frac{V_{50\%\;\mathrm{isodose}\;}}{\mathrm{PIV}}$$

Where the *PIV* is the prescription isodose volume. In our study we take the *PIV* to be the volume of tissue receiving 100% of the prescribed dose.

However, in a multi‐target plan, the global 100% and 50% isodose volumes are not sufficient to calculate target‐specific metrics. Therefore, we again adopt the definitions used by Desai *et al.* 3, for a given target PTV*_n_*:

(A8)
$$R_{50\%,\;n}=\frac{\left[\left(V_{50\%,\;\mathrm{shell},\;n}\right)+V_{\mathrm{PTV},n}\right]}{V_{\mathrm{PTV},n}}$$

(A9)
$$\;CI_{\;n}=\frac{\left[\left(V_{100\%,\;\mathrm{PTV},\;n}\right)+\left(V_{100\%,\;\mathrm{shell},\;n}\right)\right]}{V_{\mathrm{PTV},n}}$$

Where $V_{\langle X\rangle \%,\;<\mathrm{structure}>,\;n}$ represents the volume (in cubic cm) of a given structure (PTV or corresponding optimization shell) that receives X% of the prescribed dose. Again, we can also arrive at a target specific Paddick CI and GI based on the optimization shells:

(A10)
$$\;CI_{\mathrm{Paddick},\;n}=\frac{{\left(V_{100\%,\;\mathrm{PTV},\;n}\;/\;V_{\mathrm{PTV},n}\right)}^{2}}{\mathrm{CI}}$$

(A11)
$$\;GI_{\mathrm{Paddick},\;n}=\frac{V_{50\%,\;\mathrm{shell},\;n\;}+V_{\mathrm{PTV},n}}{V_{100\%,\;\mathrm{shell},n}+V_{100\%,\;\mathrm{PTV},n}}$$
